# Infant rats can learn time intervals before the maturation of the striatum: evidence from odor fear conditioning

**DOI:** 10.3389/fnbeh.2014.00176

**Published:** 2014-05-15

**Authors:** Julie Boulanger Bertolus, Chloe Hegoburu, Jessica L. Ahers, Elizabeth Londen, Juliette Rousselot, Karina Szyba, Marc Thévenet, Tristan A. Sullivan-Wilson, Valérie Doyère, Regina M. Sullivan, Anne-Marie Mouly

**Affiliations:** ^1^Lyon Neuroscience Research Center, INSERM U1028, CNRS UMR5292, University Lyon1Lyon, France; ^2^Child and Adolescent Psychiatry, Emotional Brain Institute, Nathan Kline Institute, New York University School of MedicineNew York, NY, USA; ^3^Centre de Neurosciences Paris-Sud, CNRS UMR 8195, University Paris-SudOrsay, France

**Keywords:** olfactory fear conditioning, ontogeny, memory, interval timing, striatum, respiration, freezing, infant rats

## Abstract

Interval timing refers to the ability to perceive, estimate and discriminate durations in the range of seconds to minutes. Very little is currently known about the ontogeny of interval timing throughout development. On the other hand, even though the neural circuit sustaining interval timing is a matter of debate, the striatum has been suggested to be an important component of the system and its maturation occurs around the third post-natal (PN) week in rats. The global aim of the present study was to investigate interval timing abilities at an age for which striatum is not yet mature. We used odor fear conditioning, as it can be applied to very young animals. In odor fear conditioning, an odor is presented to the animal and a mild footshock is delivered after a fixed interval. Adult rats have been shown to learn the temporal relationships between the odor and the shock after a few associations. The first aim of the present study was to assess the activity of the striatum during odor fear conditioning using 2-Deoxyglucose autoradiography during development in rats. The data showed that although fear learning was displayed at all tested ages, activation of the striatum was observed in adults but not in juvenile animals. Next, we assessed the presence of evidence of interval timing in ages before and after the inclusion of the striatum into the fear conditioning circuit. We used an experimental setup allowing the simultaneous recording of freezing and respiration that have been demonstrated to be sensitive to interval timing in adult rats. This enabled the detection of duration-related temporal patterns for freezing and/or respiration curves in infants as young as 12 days PN during odor fear conditioning. This suggests that infants are able to encode time durations as well as and as quickly as adults while their striatum is not yet functional. Alternative networks possibly sustaining interval timing in infant rats are discussed.

## Introduction

The ability to time events is continuously used in humans and other animals. It leads to the prediction of events, to the production of appropriate responses, and to the detection of errors in usual temporal patterns. It allows us, for example, to estimate if we have time to cross the street or if we have to stop when the traffic light turns yellow by estimating when it will turn red. Pavlov (1927) was the first to describe the encoding of the temporal relationships between events. Pavlovian conditioning is now defined as the pairing of an initially neutral stimulus (the conditioned stimulus, CS) with an unconditioned one (US). After repeated presentations of this association, the CS elicits conditioned responses which appear to be anticipatory to the arrival of the US (Pavlov, 1927). This suggests an encoding of the temporal relationships between the CS and the US.

The ability to perceive, estimate and discriminate durations in the range of seconds to minutes is referred to as interval timing. Very little is currently known about the ontogeny of interval timing throughout development. Fitzgerald et al. (1967) demonstrated that human infants as young as 1-month-old can be conditioned to temporal regularities. Indeed, when exposed to repeated light/dark switches, they exhibit pupillary dilatation, which becomes regular regardless if the light stimulus is presented or not. Since then, several studies have compared time judgments during development and showed that time estimation improves throughout childhood (see a meta-analysis by Block et al., 1999; a review by Droit-Volet, 2011). In animals, most of the studies carried out to investigate the ontogeny of time encoding in rats have used eyeblink conditioning, which involves interval durations in the milliseconds range. This procedure usually consists of pairing an auditory stimulus with an eyeblink-eliciting US (e.g., a mild air-puff to the eye or a mild shock to the eyelid). After many CS-US pairings (usually 100–300), a conditioned eyeblink response occurs such that the peak amplitude of the conditioned response occurs at or just before the onset time of the US (Smith, 1968; Smith et al., 1969). Eyeblink conditioning has been shown to emerge gradually between post-natal (PN) day 17 and PN24 (Stanton et al., 1992; Freeman et al., 1993). However, while interstimulus interval discrimination of the conditioned eyeblink response occurs in juvenile rats, performance shows protracted development through adulthood (Freeman et al., 2003; Brown et al., 2006). Concerning the timing of longer duration intervals (seconds to minutes range), only two studies (Lejeune et al., 1986; Lejeune, 1992) investigated interval timing in juvenile (20–30 days of age) rats using a Fixed Interval procedure, where a food reinforcer was delivered in response to lever presses on a fixed 60 s schedule. This procedure is used to investigate the ability of animals to adjust to the temporal regularities of their environment. The studies by Lejeune et al. (1986) and Lejeune (1992) reported excellent temporal regulation of behavior in juvenile rats. However, since Fixed Interval procedures require multiple training days, juvenile rats reached the age of 30 days at the end of training. To our knowledge, no experiment has been conducted to test interval timing at younger ages in rats.

The neural circuits sustaining the encoding and memorization of temporal information are still a matter of debate. Numerous structures have been suggested to be involved, with considerable differences depending on the paradigms, and no structure has been exclusively related to time encoding. Yet, there is a consensus in the literature that the areas critically involved in timing depend on the timescale considered. Indeed, the cerebellum might be crucial for sub-second durations, while the striatum and prefrontal cortex might be more involved in processing seconds to minutes interval durations (for reviews, see Buhusi and Meck, 2005; Meck et al., 2008). More specifically, a number of recent studies have demonstrated that the striatum is an important component of the interval timing system both in humans and animals (reviewed in Buhusi and Meck, 2005; Coull et al., 2011). However, anatomical studies showed that the striatum morphological maturation occurs around PN15 (Chronister et al., 1976; Tepper and Trent, 1993). Moreover, Tepper et al. (1998) reported that the electrophysiological characteristics of the striatal neurons continue to mature throughout the preweaning period. This delayed ontogenesis of the striatum raises the question of whether rats are capable of timing intervals prior to striatum development. The global aim of the present study was to investigate interval timing abilities in rats at an age for which the striatum was not yet mature.

We addressed this question using odor fear conditioning. Indeed, in comparison with the Fixed Interval procedure, fear conditioning makes very little demand on the rat motoric capabilities. Furthermore, odor fear conditioning can be applied to very young animals (Sullivan et al., 2000) as their sense of smell is fully functional at birth, contrary to vision and audition.

We first investigated whether the striatum, known for its role in time processing, was activated by odor fear conditioning in developing rats using 2-DG autoradiography. Although fear learning was shown at all tested ages, activation of the striatum was observed in adults but not in juvenile animals. Next, we assessed the presence of evidence of interval timing in ages before and after the inclusion of the striatum into the fear conditioning circuit. For this, we used an experimental setup allowing the simultaneous recording of freezing and respiration which have been demonstrated to be sensitive to interval timing in adult rats (Hegoburu et al., 2011; Shionoya et al., 2013). This enabled us to assess the emergence of temporal patterns during the acquisition of the odor-shock association in both adult and infant rats. Such analysis revealed evidence of interval timing in ages prior to and after the functional maturation of the striatum.

## Methods

### Animals

The subjects were male and female Long Evans rats born and bred either in the Nathan Kline Institute colony (originally from Harlan, USA) or in the Lyon Neuroscience Research Center (originally from Janvier, France). Only one female and one male pup per litter per treatment/test condition were used for all experiments and animals from the same litters were used in the different treatment/test conditions and ages. A total of 20 litters were used. Three groups of ages were used: PN day 12–15 (PN12–15, infants), PN22–24 (juveniles) and older than PN75 (adults). Day of birth was considered PN0. Pups were maintained with their litters up to the end of the experiments, including juvenile pups. Adults were housed by pairs at 23°C and maintained under a 12 h light-dark cycle (lights on from 6:00 am to 6:00 pm). Food and water were available *ad libitum* and abundance of wood shavings was supplied for nest building. All experiments were conducted in strict accordance with the Institutional Animal Care and Use Committee of the Nathan Kline Institute, which follows the guidelines from the American National Institutes of Health, and with the European Community Council Directive of November 24, 1984 (84/609/EEC) and the French National Committee (87/848) for care and use of laboratory animals. Care was taken at all stages to minimize stress and discomfort to the animals. An overlap in personnel conditioning/testing both infant and adult rats in France and the USA ensured consistency of conditioning and testing of infant and adult animals between labs.

### Training apparatus

Equivalent conditioning apparatus and procedures were used between labs as previously described (USA: Coulbourn equipment, described in Sevelinges et al., 2008; France: Emka and Coulbourn equipment, described in Hegoburu et al., 2011). In the USA, a standard Coulbourn shock chamber was used. In France, a custom built Plexiglas conditioning chamber for fear conditioning was used and equipped with features allowing fine-grain freezing analysis (4 camera views, B/W CMOS PINHOLE camera, Velleman, Belgium, and an homemade acquisition software using Matrox Imaging Library and acquisition card, Matrox video, UK) and a plethysmograph (diameter 30 cm, Emka Technologies, France) for measuring respiratory frequency (see Hegoburu et al., 2011 for further description of the plethysmograph). The height of the plethysmograph was adapted to the age of the animal in order to optimize the signal-noise ratio, leading to a height of 30 cm for the adults and 16.5 cm for the infants.

### Odor fear conditioning procedure

CS-US parameters were standardized between labs. The American lab used the Coulbourn FreezeFrame software for stimuli delivery control and video recording. The French lab used a custom build program for stimuli delivery and recording. Conditioning took place in a sound attenuation chamber with deodorized air constantly flowing through the cage (2 L/min). The odor CS was a 30-s peppermint odor (McCormick Pure Peppermint; 2 L/min; 1:10 peppermint vapor to air) and was controlled with a solenoid valve that diverted the airflow to the peppermint air stream, thus minimizing pressure change. The 1-s mild electric shock was delivered through a grid floor. In the US, for the youngest pups, the shock was delivered through an electrode to one hind limb. At both sites, adult rats were handled for about 4 days and placed into the conditioning chamber for context habituation. Juveniles received only 1 day of handling and habituation while infants, for which conditioning to context is not yet developed (Raineki et al., 2010), were not handled to minimize distress from separation from the mother.

Three training conditions were used throughout the experiments: Odor-shock pairings (Paired condition), Odor-shock unpaired presentation (Unpaired condition) and Odor-alone presentation (Odor group). In the paired groups, during the first 10 min of the conditioning session, the animals were allowed an adaptation period of free exploration. Then the CS odor was introduced into the cage for 30 s, the last second of which overlapped with the shock. The animals received ten odor-shock trials, with an inter-trial interval of 4 min. In the Unpaired groups, the same procedure was carried out except that the shock and the odor were explicitly unpaired using a fixed long duration (180 s) between the odor onset and the shock arrival. In the Odor groups, the odor was presented alone for 30 s.

### Retention test

A subset of animals at the three ages (Infants: *n* = 17; Juveniles: *n* = 22; Adults: *n* = 26) were tested the day after conditioning for learned fear responses. Testing was done by an experimenter blind to the training conditions.

Infants were tested in a y-maze (start box: 8.5 × 10 × 8 cm; choice arms: 8.5 × 24 × 8 cm), one arm containing the peppermint odor CS (20 μL peppermint on Kim Wipe), and the other containing familiar pine shavings (20 mL clean bedding). Pups were given 5 trials. For each trial, the pup was placed in the start box (5 s), the door to each alley opened and the animals were given 60 s to choose an arm. The number of choices of the arm containing the CS odor was compared between groups using a One-Way ANOVA with the Group as an independent factor.

For adults and juveniles, the rat was placed in a novel experimental cage with new contextual (tactile and visual) cues and allowed a 10-min odor-free period. The CS odor was then presented five times (30 s with a 4-min inter-trial interval) and the animal's freezing response was hand scored by an experimenter blind to the training conditions. The average freezing behavior of a 25 s period preceding odor delivery (Pre-CS period) was compared with the full odor presentation period (CS period) using a Two-Way ANOVA with Group (Paired, Unpaired and Odor) as an independent factor and Period (Pre-CS vs. CS) as a repeated measures factor. Significant ANOVAs were followed by *Post-hoc* Fisher comparisons.

### Striatum 2-DG autoradiography

In the ^14^C 2-DG autoradiography, the animals (adults: *n* = 17, juveniles: *n* = 18) were injected with 2-DG (20 μCi/100 g) 5 min prior to conditioning (Paired, Unpaired and Odor). Brains were removed immediately after the 45 min of conditioning, frozen in 2-methylbutane (−45°C) and stored at −70°C. Brains were then cut into 20 μm coronal sections (following equilibration to −20°C) and placed in exposure cassettes for 4 days with standards (^14^C methylmethacrylate standard 10 × 0.02 mCi; American Radiolabeled Chemicals, Inc.), scanned (Epson) and analyzed using NIH Image J Software for quantitative optical densitometry. The dorsal striatum was divided into anterior (Bregma +1.9 mm, Paxinos and Watson, 1986) and posterior dorsal striatum (Bregma +0.1). Within each part, the lateral and medial dorsal striatum were differentiated, as they receive inputs from different parts of the brain (McGeorge and Faull, 1989). Coordinates were adjusted in pups to obtain similar measure locations in both adults and pups. To control for potential differences in section thickness and autoradiograph exposure, 2-DG uptake was measured relative to 2-DG uptake in the corpus callosum that did not vary between conditioning groups (Sullivan and Leon, 1986; Sullivan et al., 2000). For each developmental age, the data from the anterior and posterior parts of the dorsal striatum were analyzed using a Three-Way ANOVA with Group as an independent factor and Anteriority (anterior vs. posterior part) and Laterality (lateral vs. medial part) as repeated measures factors. A One-Way ANOVA was then carried out on the data of each of the four subparts, followed by *post-hoc* Fisher comparisons allowed by the ANOVA results.

### Assessment of temporal patterns of freezing and respiration during acquisition

To assess interval timing during development we used olfactory fear conditioning in PN12–15 infant (*n* = 30) and adult rats (*n* = 25), ages representative of before and after functional striatal development. Three experimental groups of animals per age were trained using either 30 s (Paired 30 s group), or 20 s (Paired 20 s group) Odor-Shock interval duration, or Odor alone presentation (Odor group).

In each experimental group, the time course of respiration and behavior was monitored throughout the acquisition session. Offline, the respiratory signal was analyzed and momentary respiratory frequency was determined. The animal's freezing behavior was automatically detected using a LabView homemade software that had been validated by comparison to hand scoring by an experimenter blind to the rats' condition. Definition of the freezing at the different ages followed the methods defined by Takahashi (1992) and takes into account the immaturity of infants' musculoskeletal system. Instant respiratory frequency and freezing were averaged on a second by second basis, leading to 1-s time bins curves. The resulting individual curves were then averaged among animals of the same experimental group.

The temporal dynamics of the recorded parameters in presence of the CS odor was compared using a Two-Way ANOVA with Group as an independent factor and Time as a repeated measures factor. *Post-hoc* pairwise comparisons were then carried out when allowed by the ANOVA results. In addition in each group, a One-Way ANOVA for repeated measures was carried out to compare each CS-bin (i.e., seconds 1–19 in the Paired 20 s group and seconds 1–29 in the Paired 30 s group) to the averaged parameter value during 25 s prior the odor arrival as a baseline. *Post-hoc* within group comparisons were then carried out when allowed by the ANOVA results. For all the statistical comparisons performed, the significance level was set at 0.05.

In order to assess whether the temporal patterns of freezing and respiratory rate observed for the Paired 20 s and Paired 30 s groups are related to the CS-US interval, we assessed whether they respected the scalar property. Indeed, a remarkable property of interval timing is that the error of time estimation varies linearly with the estimated interval. This is known as the scalar property of time estimation (Gibbon, 1977). As a consequence, when the behavioral temporal response functions are normalized by the criterion time (multiplicative rescaling), they superimpose (reviewed in Matell and Meck, 2000). To test if this property was respected for the freezing and respiratory rate temporal responses, the time axis for the individual curves of the Paired 30 s rats was multiplicatively rescaled so that the 19 bins of time of CS for both groups represented the same proportions of elapsed time from CS onset to shock presentation. The scalar timing rule predicts superior superposition of the functions in relative time (multiplicative transform, comparison of the Paired 20 s average curve to the rescaled Paired 30 s average curve), compared to no rescaling (comparison of the Paired 20 s average curve to bins 1–19 of the Paired 30 s average curve) or rescaling by an additive shift of x-axes (additive transform, comparison of the Paired 20 s average curve to bins 11 to 29 of the Paired 30 s average curve). Superposition was indexed by eta-square (η^2^), a measure of the proportion of variance accounted for by the mean of the two functions (Brown et al., 1992). When superposition is perfect, η^2^ is at its maximum value of 1. Superposition was assessed on curves obtained with absolute freezing and respiratory rate values as well as with data normalized in relative response rate (maximum minus minimum values) on the Y axis.

## Results

### Fear learning: Odor-shock conditioning produced learning at all ages

**Figure 1:**
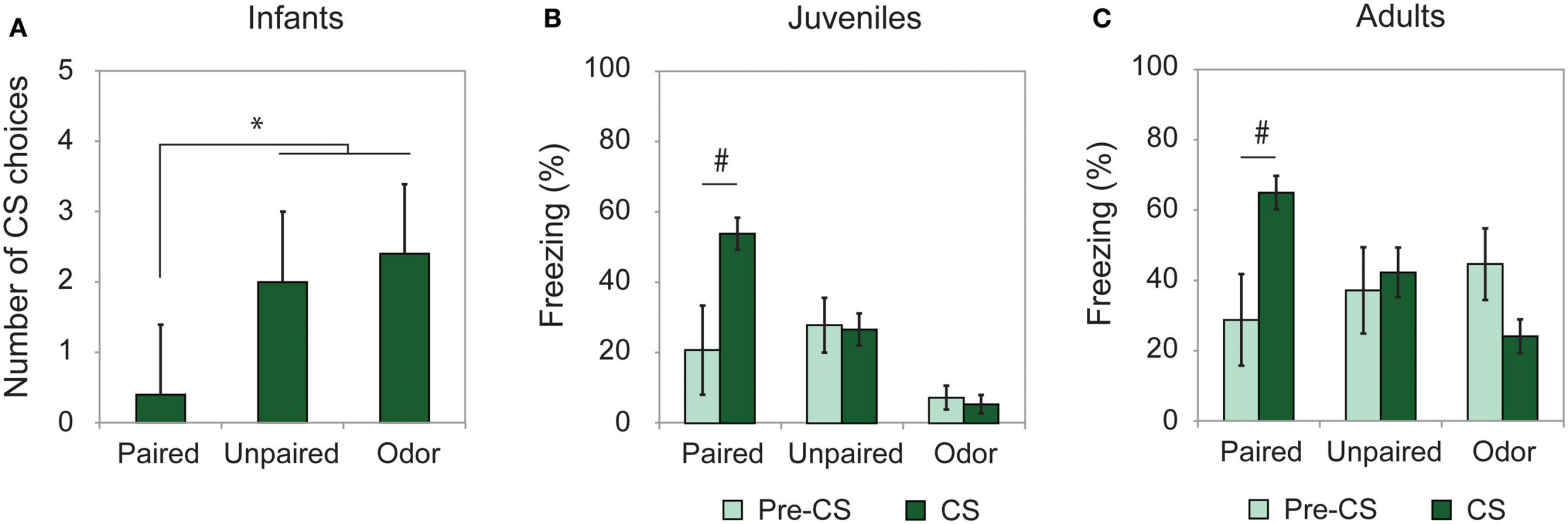
**Learning retention session**. Animals were tested the day after conditioning. **(A)** Infant rats (Paired, *n* = 5; Unpaired, *n* = 6; Odor, *n* = 6) were given a Y-Maze test and the number of CS choices (mean ± s.e.m.) out of 5 were used as a measure of learning. **(B)** Juveniles (Paired, *n* = 7; Unpaired, *n* = 9; Odor, *n* = 6) and **(C)** adults (Paired, *n* = 8; Unpaired, *n* = 8; Odor, *n* = 10) received a cue test in a novel environment and the freezing rate (mean ± s.e.m.) was compared between pre-CS and CS Odor. ^#^Significant intragroup difference (*p* < 0.05); ^*^significant intergroup difference (*p* < 0.05).

To assess learning from odor-shock conditioning at all ages, some animals were conditioned and tested for retention approximately 24 h later. As illustrated on Figure 1, all paired odor-shock conditioning animals showed learning at testing. Infants tested in a Y-Maze (Figure 1A) showed a significant Group effect [*F*_(2, 14)_ = 13.404, *p* = 0.001] and *post-hoc* Fisher analysis indicated that the paired groups were significantly different from each of the controls (Paired vs. Unpaired *p* = 0.002; Paired vs. Odor *p* < 0.001). Juvenile and adult rats received a cue test (CS presentations) in a novel context with learning assessed through freezing rate and analyzed with Two-Way ANOVA with repeated measures (Pre-CS vs. CS). Juvenile animals (Figure 1B) showed a significant Group × Period interaction [*F*_(2, 20)_ = 4.234, *p* = 0.029]. *Post-hoc* analysis evidenced a significant freezing increase during the CS odor in the Paired group (*p* = 0.02) while this increase was not present in the Unpaired and Odor groups (*p* > 0.05). Adult rats (Figure 1C) showed a significant Group × Period interaction [*F*_(2, 23)_ = 6.462, *p* = 0.006]. In the Paired group, the level of freezing was higher during the CS presentation than during the Pre-CS period (*p* < 0.05), while it remained unchanged in the Unpaired and Odor groups (*p* > 0.05).

Therefore, the training paradigm used in the present study resulted in good memory at the three developmental ages considered.

### Striatal^14^C 2-DG autoradiography: The posterior dorsal striatum is activated during odor-shock conditioning in adults but not in juveniles

**Figure 2:**
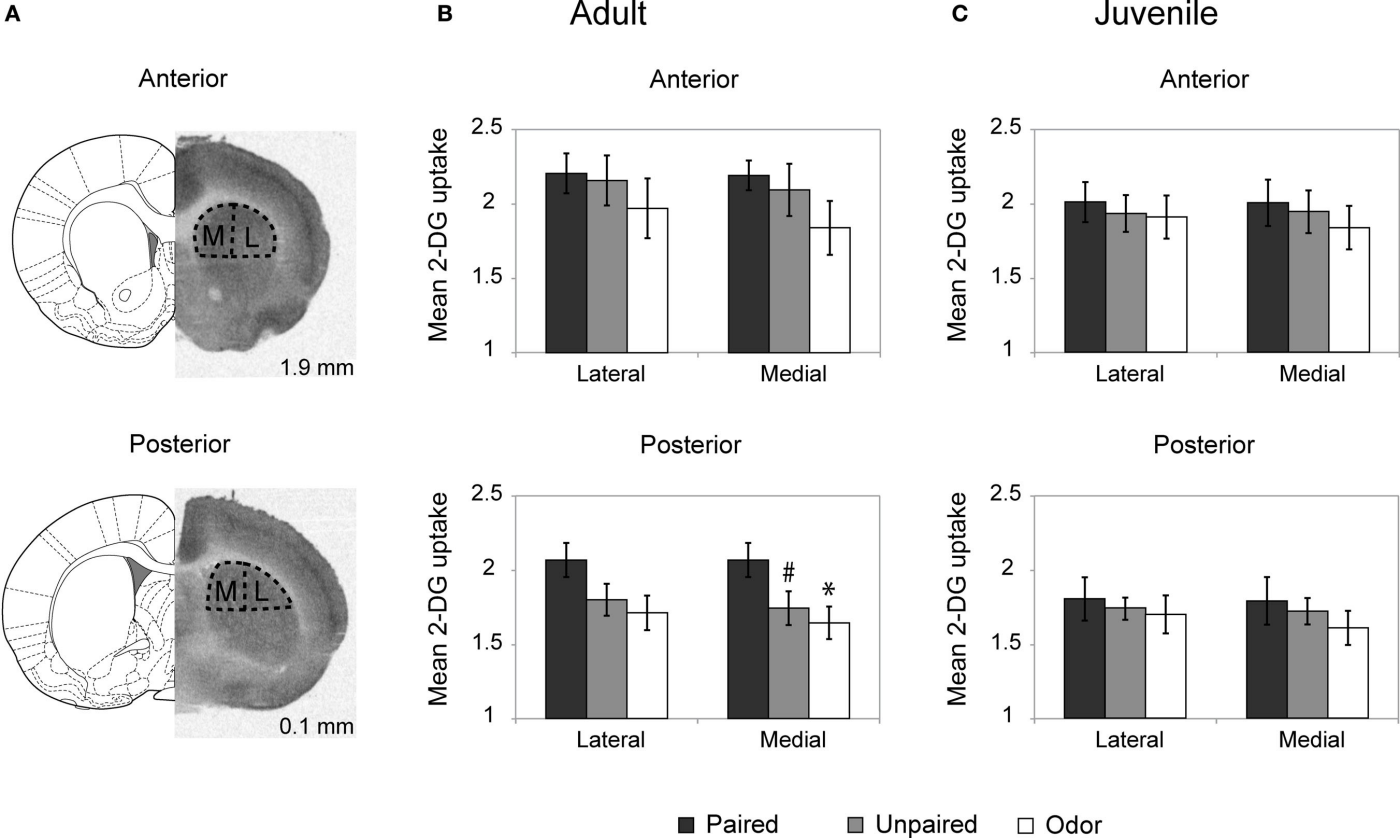
**Striatum 2-DG autoradiography during odor fear conditioning. (A)** 2-DG autoradiograph with the corresponding coronal brain sections from Paxinos and Watson (1986; numbers on the right indicate distance relative to bregma in mm). Anterior and posterior parts of the dorsal striatum were analyzed separately and subdivided in lateral (L) and medial (M) portions for optical densitometric measurements. **(B,C)** Mean 2-DG uptake ratio (±s.e.m.) in the dorsal striatum relative to corpus callosum during odor fear conditioning in adult **(B)** and juvenile animals **(C)** in the different experimental conditions: Adult Paired (*n* = 5); Adult Unpaired (*n* = 6); Adult Odor (*n* = 6); Juvenile Paired (*n* = 6); Juvenile Unpaired (*n* = 6); Juvenile Odor (*n* = 6). ^#^*p* = 0.066, ^*^p = 0.021, Fisher test comparison with the Paired group.

The level of activation of the dorsal striatum (Figure 2A) during odor fear acquisition was assessed in adults and juveniles using ^14^C 2-DG autoradiography.

In adults (Figure 2B), the Three-Way ANOVA revealed a significant effect of Anteriority [*F*_(1, 14)_ = 21.65, *p* < 0.001], Laterality [*F*_(1, 14)_ = 19.67, *p* = 0.001] and Laterality × Group interaction [*F*_(1, 14)_ = 4.51, *p* = 0.03]. Further One-Way ANOVA revealed that in the anterior part of the dorsal striatum (upper panel) the level of activation did not vary between groups for both the lateral [*F*_(2,14)_ < 1] and medial [*F*_(2, 14)_ = 1.24, *p* = 0.32] parts. In the posterior part of the striatum (lower panel), the One-Way ANOVA revealed a main effect of the Group in the medial part [*F*_(2, 14)_ = 3.62, *p* = 0.05] but no significant effect in the lateral part [*F*_(2, 14)_ = 2.50, *p* = 0.12] although the same tendency was observed. *Post-hoc* Fisher tests revealed that in the medial part, the Paired group exhibited a higher level of 2-DG uptake compared to the Odor group (*p* = 0.021) and the unpaired group (tendency, *p* = 0.066).

In juvenile rats (Figure 2C), the activation of the striatum was at a similar level between all groups regardless if the measure was taken on the anterior or the posterior dorsal striatum, medial or lateral parts (for all comparisons: *F* < 1).

In summary, in adult rats, odor fear conditioning was associated with an increased activation in the posterior part of the dorsal striatum, while in juveniles no increase was observed.

### Temporal patterns for freezing and respiration during acquisition: Infants show a temporal pattern of US expectancy

In order to assess the ability of animals of the different ages to encode interval duration in our odor fear conditioning paradigm, the temporal patterns of responses of animals conditioned to a 30 s and to a 20 s CS-US interval were compared in adult and infant rats. For both ages, we examined the temporal patterns of freezing and respiration during the CS presentation in 1-s time bins. The average curves of the last four odor-shock pairings were pooled together for each parameter (i.e., freezing and respiratory rate). Both parameters were compared between and within groups using a Two-Way ANOVA with factor Group (Paired 20 s, Paired 30 s, and Odor) and repeated factor Time. The comparison was made on the common CS duration between the Paired 20 s and Paired 30 s groups (1–19 s).

#### Adults

**Figure 3:**
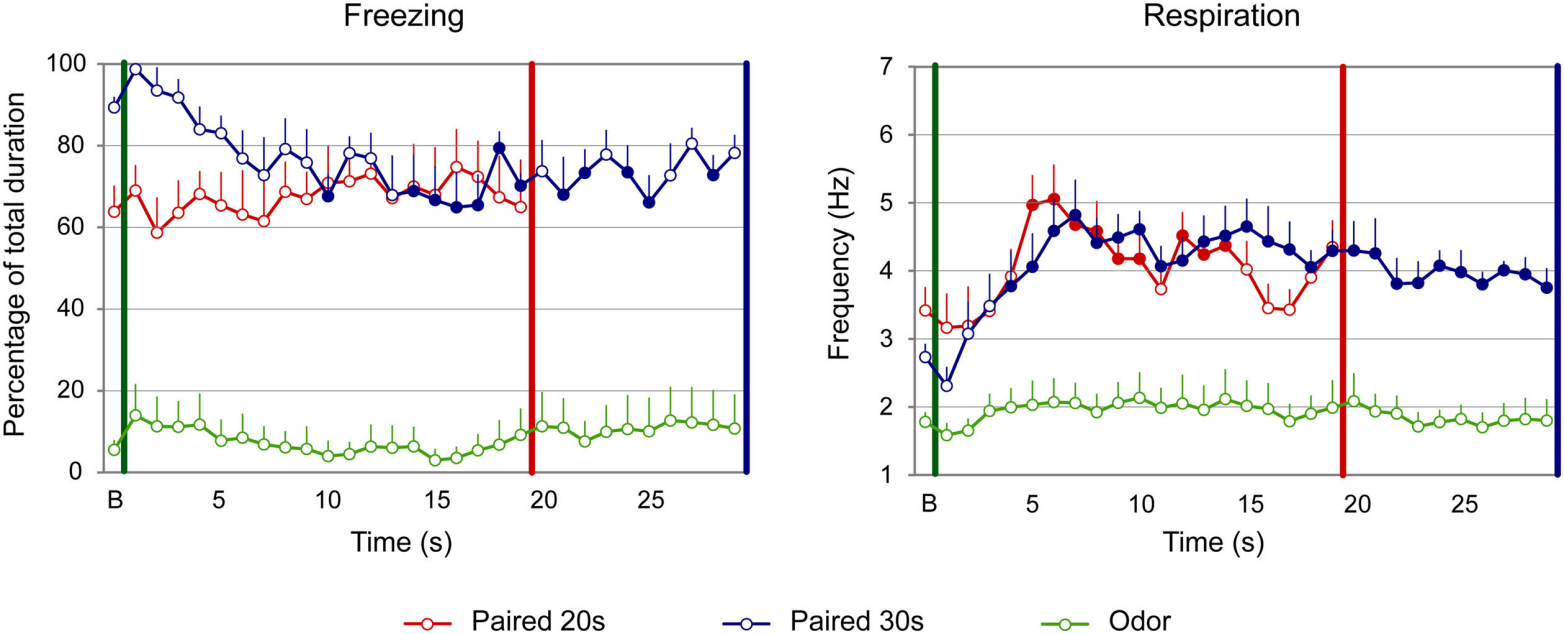
**Effect of odor-shock conditioning on fine-grain temporal pattern of freezing and respiration during odor presentation in adult rats**. The temporal pattern is represented with a 1-s bin precision, from the odor onset (green vertical line on each graph) to shock arrival (red and blue vertical line on each graph) for the paired groups. Point B on the x-axis represents the averaged baseline. Paired 20 s group: red (*n* = 8), Paired 30 s group: blue (*n* = 8) and Odor group: green (*n* = 9). Filled circles on each curve indicate the values that are significantly different from the baseline (*p* < 0.05).

For freezing (Figure 3, left panel), the ANOVA revealed a significant effect of Group [*F*_(2, 22)_ = 51.709, *p* < 0.001], Time [*F*_(18, 396)_ = 1.906, *p* = 0.014] and Group × Time interaction [*F*_(36, 396)_ = 1.831, *p* = 0.003]. Further analyses revealed that both Paired groups showed higher levels of freezing than the Odor group [Paired 20 s vs. Odor, Group: *F*_(1, 15)_ = 52.737, *p* < 0.001; Paired 30 s vs. Odor, Group: *F*_(1, 15)_ = 125.292, *p* < 0.001]. In addition, there was a significant difference between Paired 20 s and Paired 30 s groups temporal patterns during CS presentation [Paired 20 s vs. Paired 30 s, Group × Time: *F*_(18, 252)_ = 2.456, *p* = 0.001]. *Post-hoc* within group comparisons showed that, in Paired 30 s animals, introduction of the CS induced a significant decrease in freezing (at seconds 10, 14 to 19, 21, 22, and 28 of the CS, *p* < 0.05) compared to baseline. In the Paired 20 s or in the Odor group no change in freezing rate was observed in presence of the CS odor.

Concerning respiration (Figure 3, right panel), the ANOVA revealed a significant effect of Group [*F*_(2, 22)_ = 14.498, *p* < 0.001], Time [*F*_(18, 396)_ = 13.033, *p* < 0.001] and Group × Time interaction [*F*_(36, 396)_ = 3.341, *p* < 0.001]. Further analyses revealed a significantly higher respiratory rate in the Paired groups compared to the Odor group [Paired 20 s vs. Odor, Group: *F*_(1, 15)_ = 20.85, *p* < 0.001; Paired 30 s vs. Odor, Group: *F*_(1, 15)_ = 22.927, *p* < 0.001], as well as a significant difference between Paired 20 s and Paired 30 s groups temporal patterns [Paired 20 s vs. Paired 30 s, Group × Time: *F*_(18, 252)_ = 2.551, *p* = 0.001]. In addition, within-group comparisons showed that while Odor animals showed almost no reaction upon odor arrival compared to baseline due to habituation, Paired animals' respiration rate increased significantly following odor onset. In particular, in Paired 20 s animals the increase was significant from seconds 5 to 14 of the CS (*p* < 0.05), after which the rate returned to baseline value (*p* > 0.05). In Paired 30 s animals, the respiratory rate increased significantly from second 4 of the CS until the shock arrival (*p* < 0.05).

In order to assess whether the temporal patterns of freezing and respiratory rate described above for the Paired groups are related to the CS-US interval, we tested whether they respected the scalar property as explained in the Methods.

**Figure 4:**
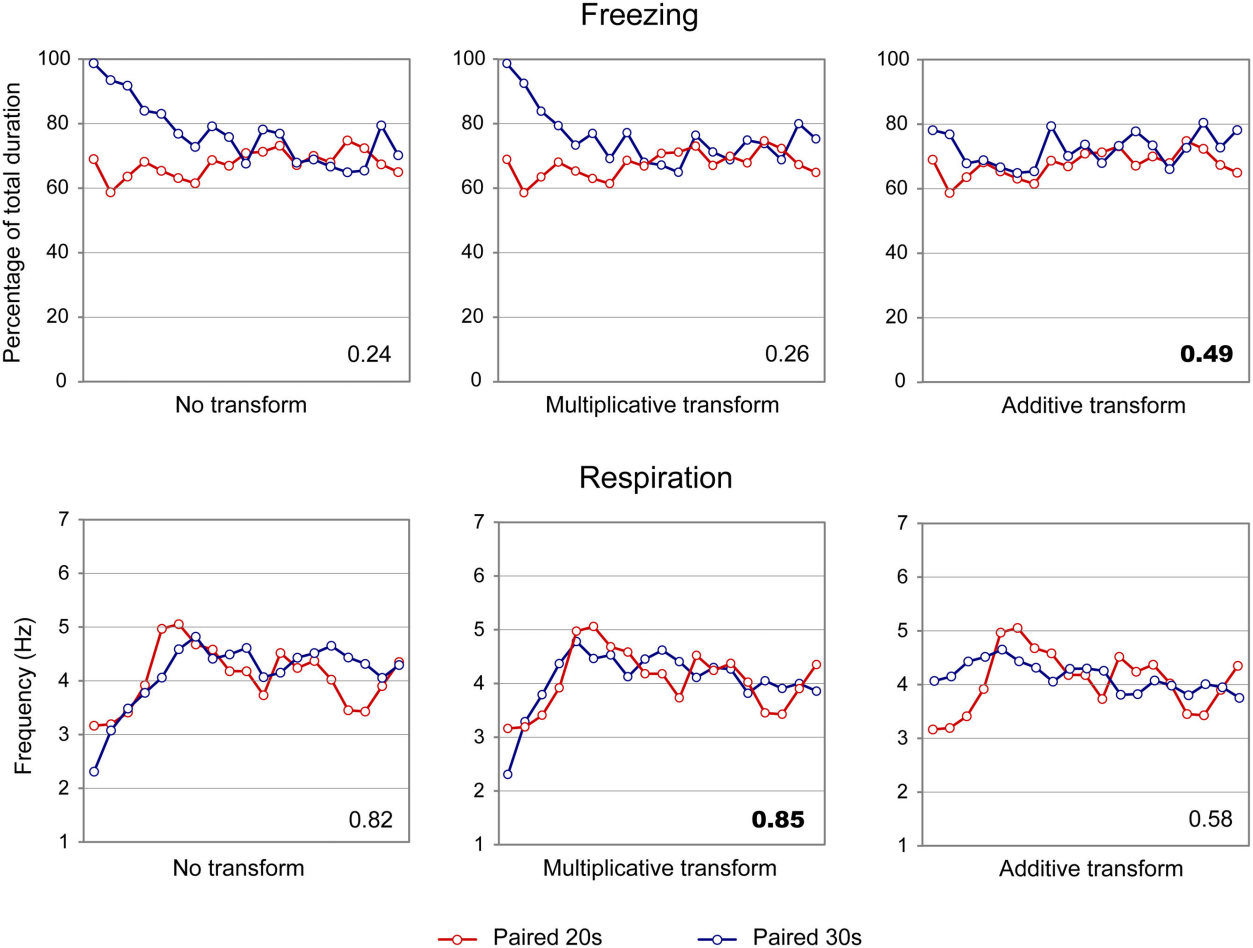
**Scalar property test on the freezing (upper part) and respiration (lower part) temporal patterns in adult rats**. The scalar timing rule predicts better superposition of the curves in relative time (multiplicative transform, **middle panel**), compared to no rescaling (**left panel**) or rescaling by an additive shift of the x-axis (**right panel**). Superposition between the two curves was indexed by eta-square (η^2^) indicated in the bottom right of each graph, the highest values being highlighted in bold characters.

When considering the freezing curves of Paired 20 s and Paired 30 s groups (Figure 4, upper part), superposition was better under the additive transform (η^2^ = 0.49, right side) than under the multiplicative transform (η^2^ = 0.26, middle) or no transform (η^2^ = 0.24, left side). In contrast, for respiration (Figure 4, lower part), superposition was better under the multiplicative transform (η^2^ = 0.85, middle) than under the additive transform (η^2^ = 0.58, right side) or no transform (η^2^ = 0.82, left side). The foregoing analyses were based on absolute response values for each dependent measure. When data were re-plotted in relative response rate on the Y axis, the results were similar, showing superior superposition with additive transform for freezing (additive: η^2^ = 0.67, multiplicative: η^2^ = 0.36, no transform: η^2^ = 0.20), and with multiplicative transform for respiration (multiplicative: η^2^ = 0.82, additive: η^2^ = 0.53, no transform: η^2^ = 0.80). Thus, the scalar property was best respected for respiration while it was not observed for freezing.

In summary, adult rats exhibited different temporal patterns of freezing and respiration rates for the two CS-US intervals. Scalar rules were respected for the respiratory rate but not for freezing, which confirms our previous data (Shionoya et al., 2013).

#### Infants

The temporal patterns of freezing and respiration rate were assessed in infants using the same analysis parameters as for adults.

**Figure 5:**
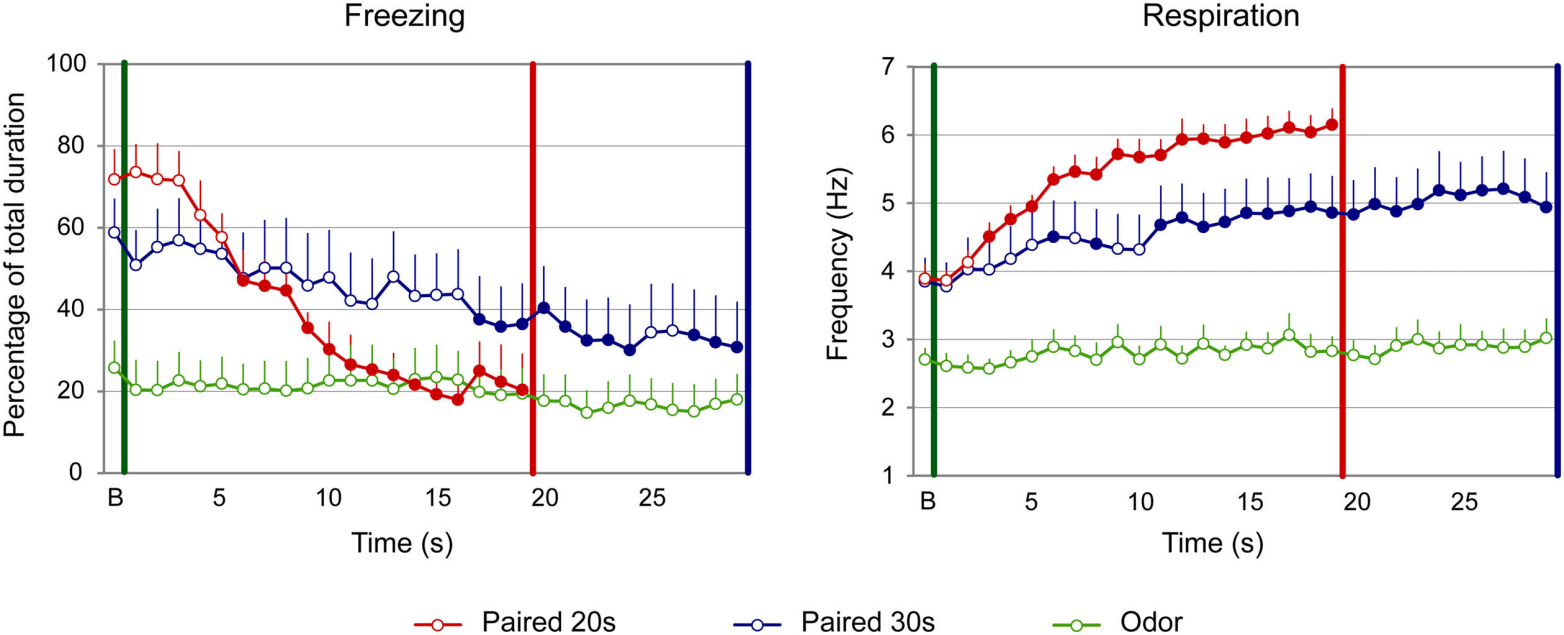
**Effect of odor-shock conditioning on fine-grain temporal pattern of freezing and respiration during odor presentation in infant pups**. The temporal pattern is represented with a 1-s bin precision, from the odor onset (green vertical line on each graph) to shock arrival (red and blue vertical line on each graph) for the paired groups. Point B on the x-axis represents the averaged baseline. Paired 20 s group: red (*n* = 10), Paired 30 s group: blue (*n* = 10) and Odor group: green (*n* = 10). Filled circles on each curve indicate the points that are significantly different from the baseline (*p* < 0.05).

Concerning freezing (Figure 5, left side), the ANOVA evidenced a tendency for a Group effect [*F*_(2, 27)_ = 3.01, *p* = 0.066], and a significant effect of Time [*F*_(18, 486)_ = 12.988, *p* < 0.001] and Group × Time interaction [*F*_(36, 486)_ = 6.656, *p* < 0.001]. Further analysis revealed that both Paired groups showed significantly different levels of freezing than Odor group [Paired 20 s vs. Odor, Group: *F*_(1, 18)_ = 4.256; *p* = 0.054; Paired 30 s vs. Odor, Group: *F*_(1, 18)_ = 4.489, *p* = 0.048]. In addition, the temporal patterns of Paired 20 s and Paired 30 s animals were significantly different [Paired 20 s vs. Paired 30 s, Group × Time: *F*_(18, 324)_ = 4.811, *p* < 0.001], although similar levels of baseline and pre-shock (3 last seconds before shock) freezing were observed in both groups (*p* > 0.05). In infants, introduction of the CS odor induced a strong decrease in freezing in the Paired groups. Indeed, within group comparisons showed that, in Paired 20 s animals, this decrease was significantly different from baseline from second 6 until shock arrival (*p* < 0.05), whereas in the Paired 30 s group significance was reached from second 17 until shock arrival (*p* < 0.05).

Concerning the respiration (Figure 5, right panel), the ANOVA revealed an effect of Group [*F*_(2, 27)_ = 17.588, *p* < 0.001], Time [*F*_(18, 486)_ = 22.209, *p* < 0.001] and of the Group × Time interaction [*F*_(36, 486)_ = 5.123, *p* < 0.001]. Further analysis evidenced that in both Paired groups the respiratory rate was significantly higher than in the Odor group [Paired 20 s vs. Odor, Group: *F*_(1, 18)_ = 98.076, *p* < 0.001; Paired 30 s vs. Odor, Group: *F*_(1, 18)_ = 10.581, *p* = 0.004]. In addition, the temporal patterns of Paired 20 s and Paired 30 s animals were significantly different [Paired 20 s vs. Paired 30 s, Group × Time: *F*_(18, 324)_ = 3.753, *p* < 0.001], although similar respiratory rates were observed at baseline and prior the shock arrival in the two groups (*p* > 0.05). Within group comparisons showed that, in the Paired 20 s group, introduction of the CS Odor induced a significant increase in respiratory rates compared to baseline from second 3 of the CS until shock arrival (*p* < 0.05), whereas in Paired 30 s animals, the increase reached significance from second 6 until the end of the CS (*p* < 0.05).

**Figure 6:**
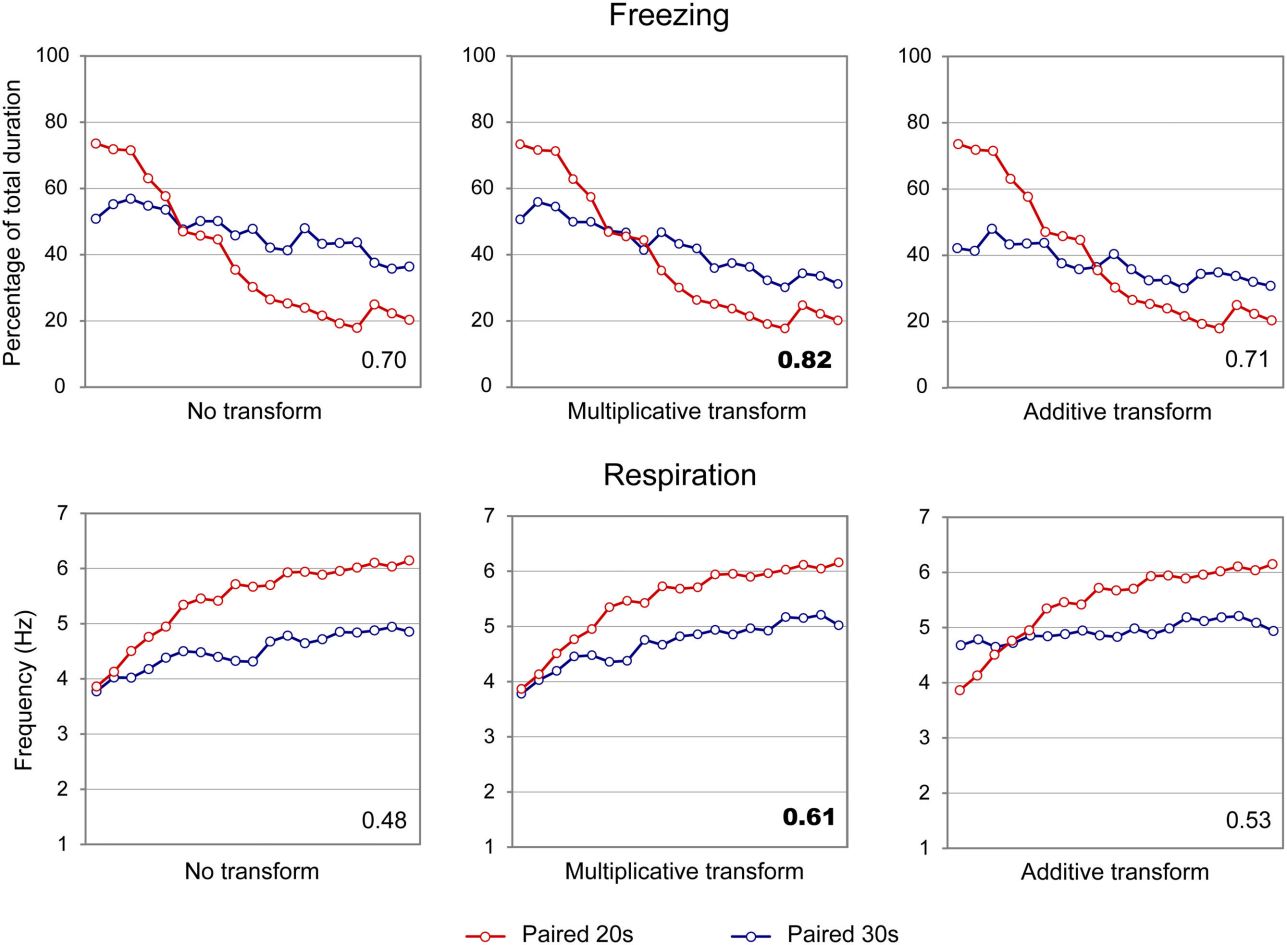
**Scalar property test on the freezing (upper part) and respiration (lower part) temporal patterns in infant rats**. The scalar timing rule predicts better superposition of the curves in relative time (multiplicative transform, middle panel), compared to no rescaling (left panel) or rescaling by an additive shift of the x-axis (right panel). Superposition between the two curves was indexed by eta-square (η^2^) indicated in the bottom right of each graph, the highest values being highlighted in bold characters.

When the scalar property was tested on the freezing curves (Figure 6, upper part), the highest superposition index was obtained for the multiplicative transform (η^2^ = 0.82, middle) compared to additive transform (η^2^ = 0.71, right side) or no transform (η^2^ = 0.70, left side). Similar results were observed for the respiratory rate curves (Figure 6, lower panel; multiplicative: η^2^ = 0.61, additive: η^2^ = 0.53, no transform: η^2^ = 0.48). When response rates were re-plotted in relative values on the Y axis, the results were similar, showing superior superposition with multiplicative transform for freezing (multiplicative: η^2^ = 0.85, additive: η^2^ = 0.78, no transform: η^2^ = 0.67), and for respiration (multiplicative: η^2^ = 0.91, additive: η^2^ = 0.63, no transform: η^2^ = 0.82). Thus, in infant rats, the scalar property was respected for both respiration and freezing.

In summary, infant rats showed different temporal patterns of freezing and respiration rates for the two duration intervals. Both freezing and respiration curves followed the scalar rules, thus supporting the hypothesis that infant pups expressed temporal learning.

## Discussion

The present study investigated the ontogeny of time durations encoding in odor fear conditioning. We first assessed whether the striatum, which is a brain area implicated in timing, was differentially activated in odor fear conditioning throughout development. This is the first study to assess striatum activity across development within this paradigm. The 2-DG metabolic mapping study revealed that, while dorsal striatum was activated during odor fear acquisition in adults, its activity remained unchanged in juveniles. These data are in agreement with data on striatum development, which indicates the juvenile striatum is still maturing (Chronister et al., 1976; Tepper and Trent, 1993; Tepper et al., 1998). To investigate interval timing abilities before and after the striatum functional maturation, we performed fine-grain analysis of behavioral and physiological responses shown to be valuable for assessment of timing in adults during the early stages of odor fear learning. Specifically, we monitored the time course of the animal's respiratory rate and freezing behavior during conditioning at two developmental ages: one with striatum activity correlated with learning (adult) and the other for which the striatum has been shown to be immature in the literature (infant). The data showed that for both adult and infant rats, duration-related temporal patterns can be detected for freezing and/or respiration curves suggesting that infants are able to encode time durations as well and as quickly as adults while their striatum is not yet functional.

### In adult animals respiration rate is a reliable index of time encoding

The present data show that in adult animals, the temporal patterns of respiration rate in the Paired 20 and 30 s groups were significantly different and followed the scalar property. In contrast, freezing temporal patterns, although different, did not respect scalar rules. These data confirm the results we obtained in a previous study using a slightly different paradigm (Shionoya et al., 2013). Indeed in that study, the same animals were first conditioned to a 20 s odor-shock interval and then shifted to a 30 s interval. This resulted in a shift of the temporal pattern of respiration toward the new duration. However, while for analytical reasons studies of timing usually manipulate the interval durations within rather than between subjects (Brown et al., 1992), this procedure may lead to biases in the temporal pattern of conditioned responses due to the previously learned duration. Here we show that differential patterns are observed when different groups of animals are used for the 20 and 30 s CS-US intervals, thus when the two groups underwent an equivalent amount of training.

The present study also confirms that, in adults, respiration is a more sensitive index than freezing to investigate the emergence of duration-related temporal patterns within a few trials (Shionoya et al., 2013). Indeed, the respiratory rate pattern respected the scalar property (Gibbon, 1977). This property refers to the observation that, in interval timing, the variability in the temporal behavior of an animal grows proportionally with the duration of the timed stimulus. This was not observed for the freezing temporal patterns suggesting that respiration is a more reliable index than freezing to assess interval timing in odor fear conditioning. This property might be due to the fact that respiratory rate is a highly fluctuant signal which can be modulated both by the sampling of odorants (Macrides et al., 1982; Youngentob et al., 1987; Kepecs et al., 2007; Wesson et al., 2008) and by the acquired emotional valence of the stimulus (Freeman et al., 1983; Monod et al., 1989; Nsegbe et al., 1997) thus allowing the observation of subtle transient variations in the animal's fear levels.

### Infant animals show interval timing abilities

In infants, both the respiration and the freezing rates showed clear temporal patterns respecting the scalar property. To our knowledge, our study is the first to show interval timing in 12–15 days old infant rats. Indeed previous studies devoted to investigate the ontogenesis of temporal learning of seconds-to-minutes intervals in rats were performed on animals aged of 21 days at the beginning of training (Lejeune et al., 1986; Lejeune, 1992). Only slightly younger ages had been investigated on the sub-second range (17-day-old: Stanton et al., 1992). The lack of data in the literature concerning younger ages can be explained by the fact that classical paradigms used to investigate interval timing in animals use peak interval procedures (Catania, 1971) or temporal discrimination tasks (Stubbs, 1968), both of which require numerous conditioning sessions and behavioral responses beyond the infants' motor abilities. In the present study, the use of fine-grain analysis of the temporal patterns of both respiration and freezing permitted us to highlight interval timing abilities occurring after only a few odor-shock pairings. This observation in rats is in line with data collected in human babies showing that they can be conditioned to temporal regularities within a few reinforced trials as early as at 1 month of age (Fitzgerald et al., 1967). In this study, Fitzgerald and colleagues used light/dark regularly spaced switches to evidence that, after about 10 switches, human infants show regular pupillary constriction or dilatation regardless if the switch occurs or not. A recent study using operant discriminative conditioning in 4-months-old babies confirmed a relatively high sensitivity to time at early ages (Provasi et al., 2011).

Interestingly in our study the use of complementary indices such as freezing and respiration permitted us to highlight parameters that revealed timing indices changes throughout development. Indeed, in infants, contrary to adults, freezing appears to be a good index of interval timing. Infant rats respond to the CS odor by a strong decrease in freezing. While it is unclear why freezing was a better measure of timing in pups compared to adults, it may be related to pups' immature freezing response (Hunt and Campbell, 1997).

### Involvement of the dorsal striatum in odor fear conditioning

The 2-DG autoradiograph revealed that, in adult Paired rats, the medial part of the posterior striatum showed an increased activity compared to Unpaired and Odor animals. A growing literature suggests the involvement of the striatum in interval timing (Allen et al., 1972; Hikosaka et al., 1989; Matell et al., 2003; Höhn et al., 2011). According to McGeorge and Faull (1989), the lateral dorsal striatum receives projections from the sensori-motor cortex, while the medial posterior dorsal striatum receives inputs from the piriform cortex (McGeorge and Faull, 1989) and the amygdala (McDonald, 1991). Therefore, the difference we observe between localizations of the striatum activation might be due to the paradigm and modalities used.

The dorsal striatum has also been suggested to be involved in aversive learning in general such as in auditory fear conditioning (Ferreira et al., 2003; Kishioka et al., 2009; Wendler et al., 2014), or in a two-way active avoidance task (Darvas et al., 2011; Wendler et al., 2014) but not in contextual fear conditioning (Ferreira et al., 2003). Interestingly, both auditory fear conditioning and two-way avoidance present some temporal regularities for the animal to learn, while contextual fear conditioning usually does not. This suggests that the striatum might be preferentially involved in time learning rather than in the learning of the aversion. This is also supported by the results in Unpaired animals of the present study, which showed a similar level of activation in the striatum as the control animals.

In juveniles, our 2-DG metabolic mapping study showed similar levels of activation in the striatum of Paired animals compared to Unpaired and Odor animals. In infants, both morphological (Chronister et al., 1976; Tepper and Trent, 1993) and electrophysiological studies (Tepper et al., 1998) suggest that the striatum is immature. Therefore, at these two developmental ages, the striatum does not seem to be involved in the learning while infant animals in our study and juvenile animals in the literature (Lejeune et al., 1986; Lejeune, 1992; Stanton et al., 1992; Freeman et al., 1993) show clear evidence of learning of interval duration. This raises the question of the neuronal network supporting timing in young animals. It has been demonstrated that neural circuits underlying learning can evolve throughout the development of the organism. The neural substrate of odor-shock associative learning, for example, changes dramatically around PN10. Prior to this age, the association is supported by the olfactory bulb and the anterior piriform cortex (Moriceau et al., 2006). After PN10, the olfactory bulb disengages (Rangel and Leon, 1995), the piriform activation switches from anterior to posterior (Roth and Sullivan, 2005) and the amygdala gets involved in the encoding of the association (Moriceau et al., 2006). Another example of modification of the neural circuit underlying a cognitive process can be found for extinction of conditioned fear. Indeed, while PN24 rats present an adult-like extinction that requires the ventromedial prefrontal cortex (vmPFC), extinction in PN17 rats does not involve the vmPFC (for a review, see Kim and Richardson, 2010). A similar switch throughout the brain maturation could be suggested to underlie time encoding in the brain. While the current model of interval timing encoding proposed in the literature requires a complex communication between the cortex and the striatum (Matell and Meck, 2004), an alternative pathway could be involved in the encoding of interval durations in pups. The olfactory cortex in particular could play a role as it is functional at birth and is known to be involved in odor fear conditioning (Sevelinges et al., 2004, 2008; Jones et al., 2007). Interestingly in a previous study we showed that glutamate and GABA release in the olfactory cortex during odor fear conditioning was correlated to the time of arrival of the CS-US trial suggesting a role for this structure in time encoding (Hegoburu et al., 2009). This is in line with data from the literature showing that sensory cortices are implicated in the processing of temporal information (Quirk et al., 1997; Shuler and Bear, 2006; Bueti et al., 2008). The amygdala could also be involved in interval timing in infants. Indeed a recent study carried out in adult rats reported that changing the CS–US interval during auditory fear memory reactivation induced a selective increase in Zif-268 activity in the lateral nucleus of the amygdala (Díaz-Mataix et al., 2013), and a growing literature suggest that the amygdala may play a role in timing the CS-US interval (Díaz-Mataix et al., 2014). As mentioned above the amygdala is involved in odor fear conditioning from the age of 10 days PN and could thus take part in time processing in infant animals. Finally, alternatively to the striatum, the olfactory tubercle could be considered. Indeed, based on its embryological, anatomical and neuro-chemical properties, this structure is considered as part of the striatum, and has been shown to be functional at early developmental ages (Alheid and Heimer, 1988; Voorn et al., 2004). A recent review by Wesson and Wilson (2011) highlighted the involvement of this structure in both basic and complex olfactory functions and its potentially critical role in interfacing sensory processing and behavioral response. Indeed the olfactory tubercle receives direct olfactory sensory input from the olfactory bulb and piriform cortex (Luskin and Price, 1983) and additional input from the olfactory amygdala (Krettek and Price, 1978; Ubeda-Bañon et al., 2007), thus enabling the association of a given olfactory stimulus with its learned emotional valence. Therefore, although the involvement of the olfactory tubercle in timing has not been investigated yet, this structure could act as a short pathway to link perception with the production of temporally structured actions, specifically at early developmental ages.

In conclusion, the present study shows that in odor fear conditioning, interval durations are learned after only few trials from a very early age. Although the underlying neural network remains to be elucidated, and may evolve with ontogeny, these findings support the hypothesis of a simultaneous encoding of the associative link between two events together with their temporal relationships (Balsam and Gallistel, 2009; Balsam et al., 2010). While further experiments are needed to assess temporal learning at earlier ages than those used in the present study, our data suggest that associative and temporal learning might be two sides of the same coin.

### Conflict of interest statement

The authors declare that the research was conducted in the absence of any commercial or financial relationships that could be construed as a potential conflict of interest.
